# Evaluation of the diagnostic accuracy of Computer-Aided Detection of tuberculosis on Chest radiography among private sector patients in Pakistan

**DOI:** 10.1038/s41598-018-30810-1

**Published:** 2018-08-17

**Authors:** Syed Mohammad Asad Zaidi, Shifa Salman Habib, Bram Van Ginneken, Rashida Abbas Ferrand, Jacob Creswell, Saira Khowaja, Aamir Khan

**Affiliations:** 1 Community Health Solutions, Karachi, 74000 Pakistan; 2 Radboud University Medical Center, 6525 GA Nijmegen, Netherlands; 3 London School of Hygiene and Tropical Medicine, London, WC1E 7HT United Kingdom; 4 StopTB Partnership, 1214 Geneva, 1214 Vernier, Switzerland; 5 Interactive Research & Development, Karachi, 75190 Pakistan

## Abstract

The introduction of digital CXR with automated computer-aided interpretation, has given impetus to the role of CXR in TB screening, particularly in low resource, high-burden settings. The aim of this study was to evaluate the diagnostic accuracy of CAD4TB as a screening tool, implemented in the private sector in Karachi, Pakistan. This study analyzed retrospective data from CAD4TB and Xpert MTB/RIF testing carried out at two private TB treatment and diagnostic centers in Karachi. Sensitivity, specificity, potential Xperts saved, were computed and the receiver operator characteristic curves were constructed for four different models of CAD4TB. A total of 6,845 individuals with presumptive TB were enrolled in the study, 15.2% of which had MTB + ve result on Xpert. A high sensitivity (range 65.8–97.3%) and NPV (range 93.1–98.4%) were recorded for CAD4TB. The Area under the ROC curve (AUC) for CAD4TB was 0.79. CAD4TB with patient demographics (age and gender) gave an AUC of 0.83. CAD4TB offered high diagnostic accuracy. In low resource settings, CAD4TB, as a triage tool could minimize use of Xpert. Using CAD4TB in combination with age and gender data enhanced the performance of the software. Variations in demographic information generate different individual risk probabilities for the same CAD4TB scores.

## Introduction

Tuberculosis (TB) remains a major cause of morbidity and mortality globally. In 2015, there were an estimated 10.4 million incident cases of TB and 1.8 million TB deaths^1^. Active case finding programs are being increasingly utilized to reduce the case-detection gap^2,3^.

In recent years, there has been growing interest in the use of chest x-rays (CXR) as a screening tool for TB within active and enhanced case finding programs^4^. Recent TB prevalence surveys have shown that CXR has higher sensitivity than verbal screening for identifying pulmonary TB^5–7^. Previously, costs, limited access to x-ray facilities, maintenance of equipment, availability of trained personnel, poor specificity and inter-observer variation meant that the role of CXR within diagnostic algorithms was limited^8^.

The advent of digital chest radiography along with software capable of automated interpretation such as the “Computer Assisted Diagnosis for TB” (CAD4TB) software developed by the Diagnostic Image Analysis Group of the Radboud University Medical Centre has prompted reconsideration of the role of CXR in TB screening, particularly in low resource, high-burden settings^9^. Long-term use of digital radiography is cost-efficient compared to conventional radiography as it eliminates recurring costs related to reagent use and radiologists^10^. Currently, CAD4TB is the only scoring software that has been evaluated and is being implemented in programmatic settings. Encouraging findings on the diagnostic accuracy of CAD4TB has been reported from sub-Saharan Africa, and most recently from Bangladesh^11–15^.

The need for improved approaches for screening has acquired greater pertinence following the introduction of sensitive rapid molecular diagnostics for TB such as Xpert MTB/RIF (Xpert) testing^16–18^. However, the scale-up of Xpert testing is limited in resource-constrained countries by high costs of test cartridges^19–22^.

An increasing body of evidence from high burden countries suggests that the use of digital CXR equipment and the automated reading of CXR with Computer Aided Detection (CAD), as a pre-screening tool, in conjunction with an expensive molecular test such as Xpert can improve case finding efforts^23^.

The use of CAD4TB is still in development phase, and the World Health Organization (WHO) has not developed any formal guidelines or recommendations for its use due to limited evidence. The aim of this study was to evaluate the diagnostic accuracy of CAD4TB as a screening tool, in Karachi, Pakistan, a megacity with a high TB prevalence and a substantial burden of undiagnosed TB. Similar studies, reporting diagnostic accuracy using Xpert MTB/RIF as the reference standard have been reported from Zambia in 2013 and Bangladesh in 2017^13,14^. Other studies from Zambia, Tanzania, South Africa and England have evaluated CAD4TB against the reference standard of culture^15,24–26^. Our current study is another data point in the series of studies, carried out in Pakistan. In addition, we also investigated whether different models of CAD4TB implementation that included routinely collected programmatic data such as age and gender can potentially enhance the diagnostic accuracy of the software and yield of TB case-detection.

## Methods

### Study design and setting

Pakistan has the fifth highest burden of tuberculosis in the world and the third largest number of undiagnosed TB cases^1^. Of the estimated 510,000 new TB cases, only 331 809 (65%) were notified to the National Tuberculosis Program (NTP) in 2015, making increased case-detection and notification a key priority^27^. Currently, smear microscopy is predominantly used as a diagnostic test in a majority of facilities in Pakistan^28^.

The study was conducted at two purpose built TB treatment and diagnostic centers, called “*Sehatmand Zindagi”* (Healthy Life) centers, in Karachi, Pakistan, from October 2013 to September 2015. These centers are located in low-middle income neighborhoods of Karachi, *Nazimabad* and *Korangi*. In addition to digital CXR equipment with CAD4TB, Xpert testing was carried out at both centers, with initiation of treatment among those diagnosed with TB.

The study was embedded within a broader programme implementing enhanced case finding, whereby community-health workers screened all individuals attending private health providers’ clinics, in the vicinity of the centers, using the WHO TB symptom screen^29^, that is screening for the presence of either of the following: cough of any duration, fever, hemoptysis, night sweats, weight loss. Following a clinical evaluation by the health providers, those identified with presumptive TB were referred to the centers for further investigation. The target population for this study included individuals with presumptive TB referred by the private providers from the catchment area of the centres, as well as individuals with symptoms who self-referred for investigation for TB. All participants underwent a paid digital CXR (USD 3–5) and were requested to provide a sputum sample for free of cost Xpert testing.

### Chest X-Ray scoring procedures

The CXRs were scored for abnormalities suggestive of pulmonary TB by a software system CAD4TB (version 3.07, Diagnostic Image Analysis Group, The Netherlands). CAD4TB was developed utilizing machine learning methods and was trained using labeled samples to differentiate between normal and abnormal x-ray images. The software has two abnormality detection systems that is textural abnormality and shape abnormality systems, which analyze the abnormalities in the unobscured lung fields that have been segmented automatically. The software then uses outputs from its detection systems as image descriptive features to train a k-NN classifier to compute a cumulative abnormality score (Range 0–95) for each CXR^13,30^. A higher score is indicative of more serious abnormality suggestive of TB. A CAD4TB threshold score of 50 was used for this population determined using previously collected CXR data in a similar population. All individuals with high CAD4TB scores (50 or greater) were referred back to their consulting physicians for further clinical evaluation.

### Data management and analysis

All individuals attending the TB centers were registered online using an open-source platform (Open MRS), by allocation of a unique patient ID, against which baseline information and history of presenting symptoms were recorded. Distribution of CAD4TB scores was compared for various patient characteristics such as age, gender, symptoms and Xpert result. Sensitivity, specificity, positive predictive value (PPV) and negative predictive value (NPV) were calculated for each of the TB symptoms using Xpert result as the standard. Univariate and multivariate associations of CAD4TB score, age, gender and symptoms (as explanatory variables) with TB infection (defined as a positive Xpert result) were computed. Logistic regressions were performed with MTB detection as the outcome variable and CAD4TB score, age and gender as the explanatory variables (Model 1 and 3). Adjusted analyses were subsequently performed through backward step-wise multivariate logistic regression using Akaike’s Information Criteria (AIC) to select the final, parsimonious model where symptoms where included as predictors of TB (Model 2 and 4). The AIC is an estimator that provides the relative quality of various statistical models and allowed for the selection of the most suitable set of predictor variables for the final model. Inclusion of the full set of symptoms screened was selected through the AIC for Models 2 and 4. Receiver Operator Characteristic (ROC) curves were constructed for four prediction models for TB, namely: Model 1 (CAD4TB score only), Model 2: (CAD4TB score, symptoms), Model 3: (CAD4TB score, Age, Gender) and Model 4 (CAD4TB score, age, gender, symptoms). Area-Under the Curve (AUC) statistics were obtained for each ROC curve and confidence intervals were calculated to investigate statistical differences in discriminatory accuracy of the prediction models. Sensitivity, Specificity, PPV and NPV for CAD4TB cutoff thresholds at scores of 50, 80 and 90 were obtained for the four prediction models by determining their predicted probabilities for TB detection. These cut offs were selected based on the CAD4TB score distribution of the study population, with score 50, 80 and 90 being at the 25^th^, 50^th^ percentile 75^th^ percentile approximately.

A range of predicted probabilities for each CAD4TB score were obtained from the two models that included CAD4TB with demographic information (age and gender) and symptoms. Locally weighted regressions were carried out for the range of predicted probabilities for both models against CAD4TB scores and were used to determine the corresponding predicted probability for MTB detection at the four CAD4TB cut-offs. Predicted probabilities of TB were computed at each CAD4TB cutoff threshold. These estimated the risk of TB detection at each CAD4TB score. These were used to estimate the number of TB cases missed, Xpert cartridges reduced (due to reduced number of individuals with a CAD4TB score above the threshold) and yield (number of MTB positive results out of all those tested) on Xpert test for the four models. All data analysis was carried out using STATA Statistical Software (Stata Corporation Version 11. College Station, TX, USA).

### Ethical Approval and informed consent

Ethical approval for the study obtained from the Institutional Review Board (IRB) of Interactive Research & Development that is registered with the Department of Health and Human Services, USA. The methods were carried out in accordance with the relevant guidelines and regulations. Verbal informed consent was obtained from the participants before carrying out screening activities under the project. De-identified data was provided for analysis to the study researchers, whereas all patient screening and diagnostic information was secured on a password-protected server.

## Results

**Figure 1:**
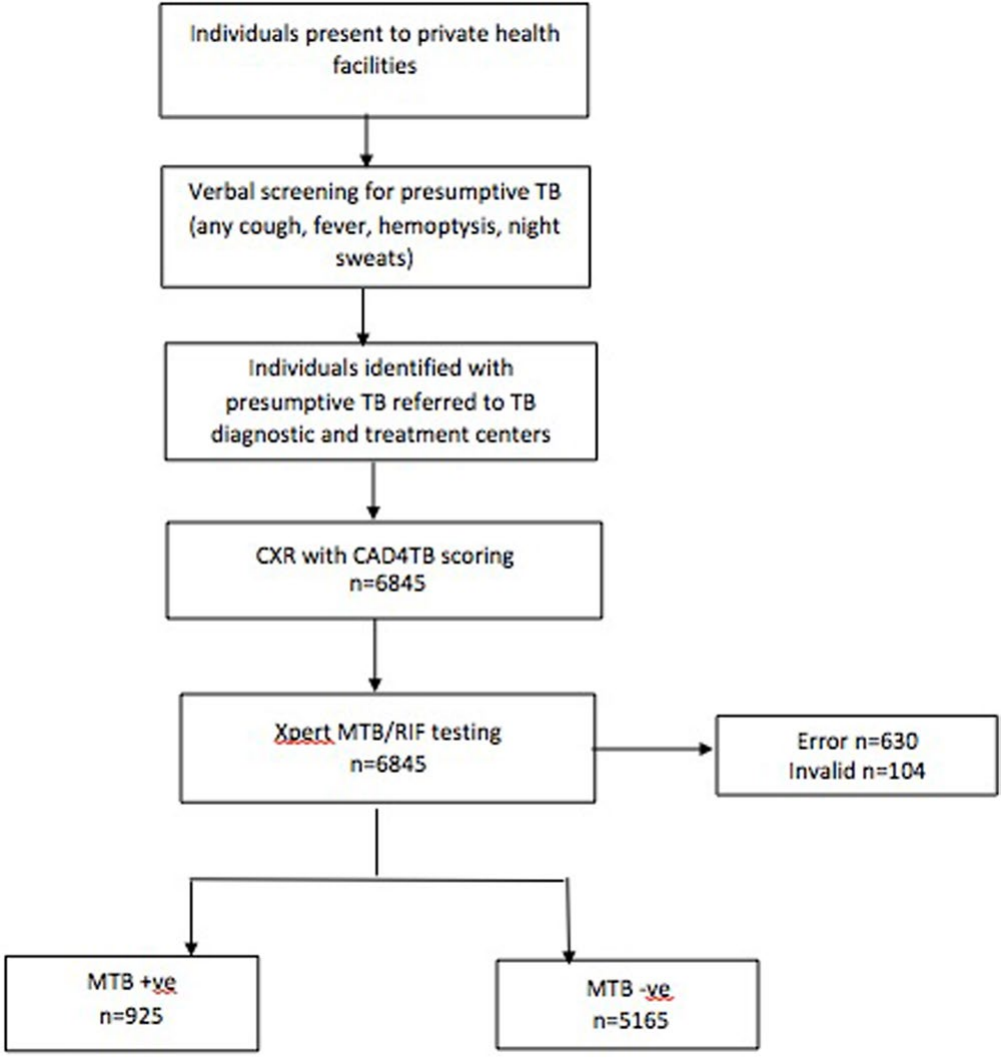
Screening algorithm. Screening and diagnostic algorithm employed for people with presumptive TB visiting TB diagnostic and treatment centers in Karachi, Pakistan (Q3- 2013 to Q2- 2015).

A total of 6,845 individuals with presumptive TB were enrolled in the study between October 2013 to September 2015. Out of these, 755 individuals, with invalid, error, no result were excluded from the analysis. The median age of participants was 38.9 (IQR 17.2) years and 3,018 (49.6%) were male. The majority of individuals included in the study reported symptoms of cough (87.5%) and fever (76.1%) (Table 1). Hemoptysis and nightsweats were reported in 13.2% and 30.5% of the study participants respectively. A total of 925 individuals enrolled in the study (15.2%) had MTB + ve results on Xpert (Fig. 1). The majority of (90.2%) people with a MTB + ve result on Xpert had a CAD4TB score >80. However, a high proportion of individuals (74.2%) that tested as MTB-ve also had scores >80 (Table 1).

**Table 1:** Baseline characteristics of individuals with presumptive TB by Computer-Aided Detection of TB (CAD4TB) scores, visiting TB diagnostic and treatment centers in Karachi, Pakistan (Q3- 2013 to Q2- 2015).

	CAD4TB scores						
	All N (%)	<=20 n (%)	21–40 n (%)	41–60 n (%)	61–80 n (%)	81–95 n (%)	p-value^*^
Gender							<0.001
Male	3,018(49.6)	51(31.7)	222(40.9)	421(41.0)	549(48.7)	1,775(54.9)	
Female	3,072(50.4)	110(68.3)	321(59.11)	605 (59.0)	578 (51.3)	1,458(45.1)	
Age							<0.001
<=20	852(14)	24(14.9)	90(16.6)	188(18.3)	177(15.7)	373(11.5)	
21–40	2,591(42.6)	101(62.7)	295(54.3)	537(52.3)	489(43.4)	1,169(36.2)	
41–60	1,732(28.4)	33(20.5)	133(24.5)	246(24)	343(30.4)	977(30.2)	
>60	915 (15.0)	3(1.9)	25(4.6)	55(5.4)	118(10.4)	712(22.1)	
**Symptoms**							
Cough							0.59
No	761(12.5)	17(10.6)	69(12.7)	176(17.2)	162(14.4)	337(10.4)	
<2 weeks	4,968(81.6)	136(84.5)	445 (82)	796 (77.6)	902(80.0)	2,689 (83.2)	
>2 weeks	361(5.9)	8(5)	29(5.34)	54(5.3)	63(5.6)	207(6.4)	
Fever							<0.001
No	1,455(23.9)	43(26.7)	139(25.6)	283(27.6)	305(27.1)	685(21.2)	
Yes	4,635(76.1)	118(73.3)	404(74.4)	743(72.4)	822(72.9)	2,548(78.8)	
Hemoptysis							<0.001
No	5,287(86.8)	134(83.2)	480(88.4)	912(88.9)	1,004(89.1)	2,757(85.3)	
Yes	803(13.2)	27(16.8)	63(11.6)	114(11.1)	123(10.9)	476(14.7)	
Night sweats							<0.01
No	4,232(69.5)	112(69.6)	379(69.8)	730(71.2)	831 (73.7)	2,180 (67.4)	
Yes	1,858(30.5)	49(30.4)	164(30.2)	296(28.9)	296 (26.3)	1,053 (32.6)	
Xpert MTB/RIF result							<0.001
MTB not detected	5,165(84.8)	159(98.8)	535(98.5)	1,003(97.8)	1,069 (94.9)	2,399(74.2)	
MTB detected	925(15.2)	2(0.2)	8(1.5)	23(2.2)	58(5.1)	834(25.8)	

(N = 6090). ^*^Significance testing was done using the chi-squared test.

Cough <2 weeks (OR 2.05, CI 1.51–2.81) was the strongest predictor of TB disease in the final adjusted models for MTB detection (Table 2). Increasing age (OR 0.96, 95% CI: 0.96–0.97) and female gender were inversely associated with a positive Xpert result (OR 0.79, 95% CI: 0.68–0.93).

**Table 2:** Predictors for TB detection among individuals tested using Xpert MTB/RIF, visiting TB diagnostic and treatment centers in Karachi, Pakistan (Q3- 2013 to Q2- 2015).

Explanatory Variable	OR^*^	95% CI	p-value^*^	Adjusted OR^*^	95% CI	p-value^*^
Age	0.98	0.97–0 0.98	<0.01	0.96	0.96–0.97	<0.01
Gender (reference group male)	0.90	0.78–1.03	0.13	0.79	0.68–0 0.93	0.004
CAD4TB Score (reference score 0)	1.08	1.08–1.09	<0.01	1.09	1.09–1.10	<0.01
**Symptoms^**						
**None (reference)**						
**Cough**						
<2 weeks	2.58	1.94–3.45	<0.01	2.05	1.51–2.81	<0.01
>2 weeks	3.00	2.03–4.40	<0.01	2.04	1.34–3.12	0.001
Fever	2.07	1.7–2.51	<0.01	1.47	1.18–1.82	<0.01
Hemoptysis	1.57	1.30–1.89	<0.01	1.35	1.09–1.67	0.005
Night sweats	1.49	1.29–1.73	<0.01	1.22	1.04–1.44	0.017

N = 6090. ^*^Significance testing has been done using chi-squared test.

^^^Symptoms were coded as binary variables.

**Figure 2:**
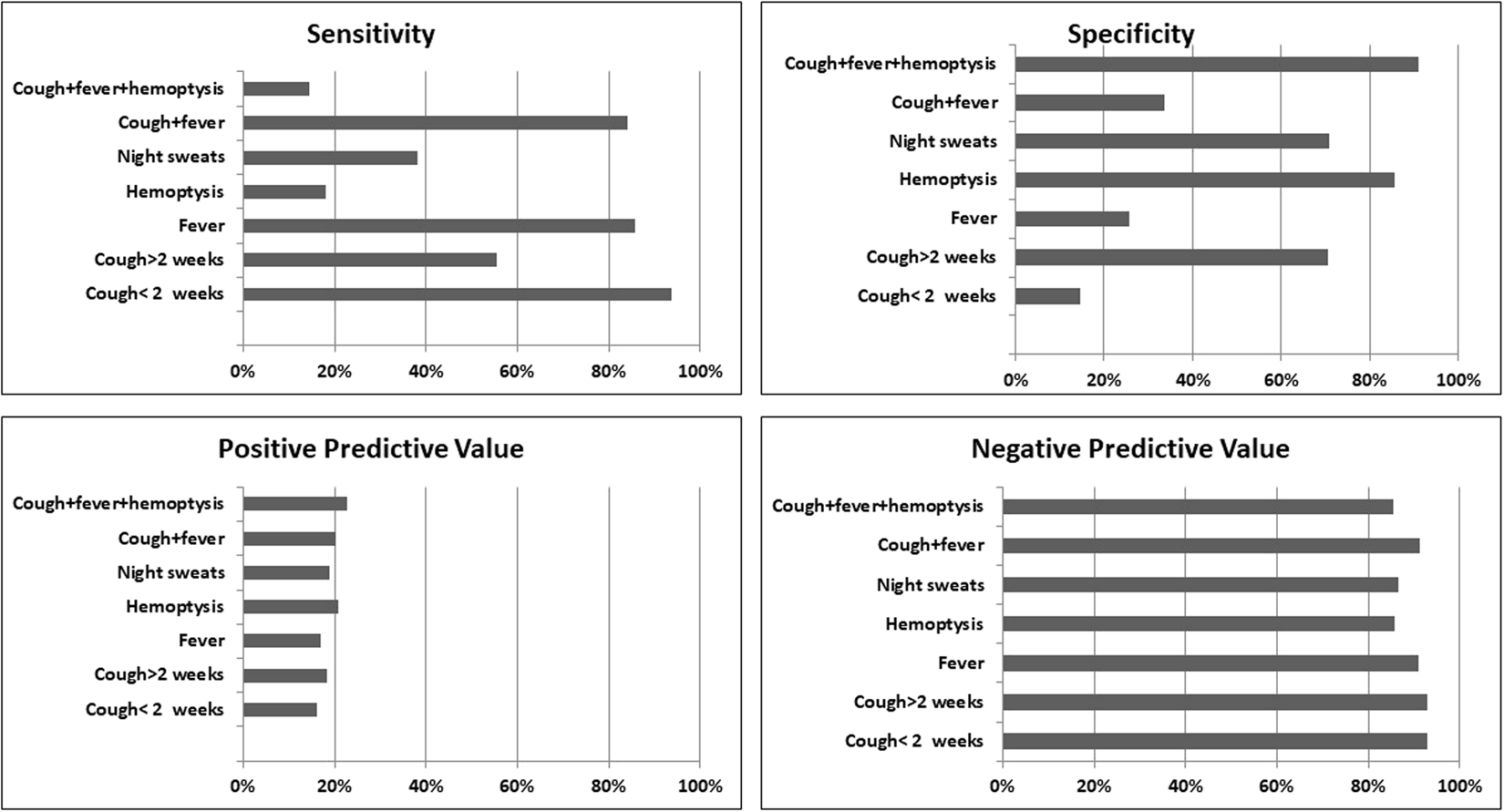
Symptomatic screening. Sensitivity, Specificity, Positive Predictive Value and Negative Predictive Value of symptomatic screening for TB of patients tested using Xpert MTB/RIF, visiting TB diagnostic and treatment centers in Karachi, Pakistan (Q3–2013 to Q2–2015).

A high sensitivity (range 65.8–95.3%) and NPV (range 93.1–98.4%) were recorded for CAD4TB (Table 3). For each model, increasing CAD4TB score thresholds, improved yield of TB case detection, with corresponding increase in specificity and decrease in sensitivity. Using the symptom screen alone, cough of <2 weeks and fever, had higher sensitivities (93.8% and 85.7% respectively) and lower specificities (14.5% and 25.6% respectively) compared to other symptoms (Fig. 2). All symptoms had high negative predictive values and low positive predictive values (Fig. 2).

**Table 3:** Sensitivity, Specificity, Positive predictive Value, Negative Predictive Value at different CAD4TB score thresholds among individuals tested using Xpert MTB/RIF, visiting TB diagnostic and treatment centers in Karachi, Pakistan (Q3–2013 to Q2–2015).

CAD Score	Sensitivity	Specificity	PPV	NPV	Xpert tests saved	Total Xpert tests	TB Cases Missed	MTB+	MTB Yield
**No Triage Test**									
—	—	—	—	—	—	**6,090**	—	**925**	**15.2%**
**Model 1: CAD4TB Only (AUC 0.79, 95% CI: 0.78–0.81)**									
50	97.3%	30.3%	20.0%	98.4%	1590	4500	25(2.7%)	900(97.3%)	20.0%
80	91.0%	50.7%	24.9%	96.9%	2702	3388	83(9.0%)	842(91.0%)	24.9%
90	85.0%	65.8%	30.8%	96.1%	3539	2551	139(15.0%)	786(85.0%)	30.8%
**Model 2: CAD4TB, Symptoms (AUC 0.81, 95% CI: 0.79–0.82)**									
50	96.75%	30.37%	19.92%	98.12%	1601	4489	30 (3.2%)	894(96.8%)	19.9%
80	87.45%	61.40%	28.85%	96.47%	3289	2801	116(12.5%)	808(87.5%)	28.8%
90	73.05%	75.75%	35.03%	94.01%	4163	1927	249(26.9%)	675(73.1%)	35.0%
**Model 3: CAD4TB, Age, Gender (AUC 0.83, 95% CI: 0.82–0.85)**									
50	96.3%	34.8%	20.9%	98.1%	1829	4261	34(3.7%)	891(96.3%)	20.9%
80	82.3%	66.9%	30.8%	95.5%	3620	2470	164(17.7%)	761(82.3%)	30.8%
90	65.8%	82.5%	40.2%	93.1%	4577	1513	316(34.2%)	609(65.8%)	40.2%
**Model 4: CAD4TB, Age, Gender, Symptoms (AUC 0.84, 95% CI: 0.82–0.85)**									
50	95.8%	37.5%	21.5%	98.0%	1973	4117	39(4.2%)	886(95.8%)	21.5%
80	82.8%	68.5%	32.0%	95.7%	3695	2395	159(17.2%)	766(82.8%)	32.0%
90	69.1%	80.9%	39.3%	93.6%	4465	1625	286(30.9%)	639(69.1%)	39.3%

For each of the models, at higher CAD4TB scores the number of Xpert tests carried out was reduced, however, it led to more patients being classified as false-negatives (TB cases missed). At a CAD4TB score of 90, a total of 3,539 Xpert tests will be saved using Model 1 (CAD4TB scores only), 4163 with Model 2 (CAD4TB scores and symptoms), 4,577 will be saved in Model 3 (CAD4TB scores, age and gender), and 4,465 in Model 4 (CAD4TB scores with age, gender and symptoms). The TB cases missed were lowest for a CAD4TB score of 50, 2.7%, 3.2%, 3.7% and 4.2% respectively for the four models. The MTB yield at a score of 90 using the four models was 30.8%, 35%, 40.2% and 39.3% respectively.

**Figure 3:**
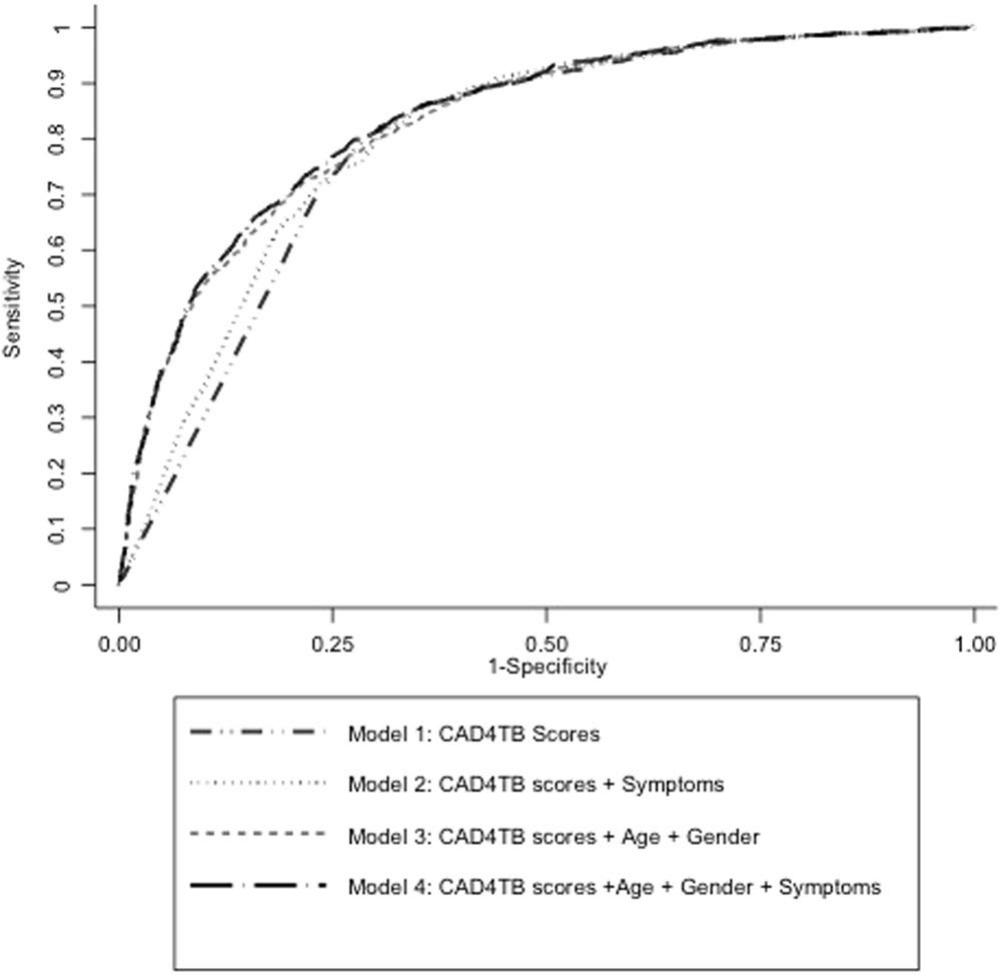
Diagnostic accuracy of CAD4TB. ROC curves yielded by the models evaluated in this study. The Area under the ROC curve (AUC) for the model with only CAD4TB scores as predictor for MTB detection (Model 1) was 0.79 (95% CI: 0.78–0.81). Model 2 (CAD4TB scores and symptoms) and model 3 (CAD4TB + symptoms + age + gender) yielded AUC of 0.81 (0.79–0.82) and 0.83 (95% CI: 0.82–0.85) respectively. Combined model using of symptoms, CAD4TB scores and age and gender (Model 4) yielded AUC of 0.84 (95% CI: 0.82–0.85).

The Area under the ROC curve (AUC) for the model with only CAD4TB scores as predictor for MTB detection (Model 1) was 0.79 (95% CI: 0.78–0.81) (Fig. 3) and for Model 2 using CAD4TB scores and symptoms was 0.81. Inclusion of patient demographics (age and gender) to CAD4TB scores (Model 3) increased the AUC to 0.83 (95% CI: 0.82–0.85). A combined model of CAD4TB scores, symptoms, age and gender (Model 4) further increased the AUC to 0.84 (95% CI: 0.82–0.85), however this was not significantly different compared to Model 3.

Table 4 describes a sample of the predicted probabilities for various combination of age and gender for the same selected CAD4TB scores.

**Table 4:** Sample of probabilities and risk for TB from a prediction model utilizing Computer Aided Detection for TB (CAD4TB) and demographic data from individuals visiting TB diagnostic and treatment centers, in Karachi (Q3 2013–Q2 2015).

CAD4TB score	Gender	Age	Predicted Probability for MTB Detection^*^	Risk for TB^**^
50	F	51	0.004	Low
50	M	51	0.005	Low
50	M	32	0.008	Low
50	F	32	0.01	Low
50	M	21	0.012	Low
50	F	21	0.016	Low
80	M	56	0.051	Low
80	F	56	0.065	Low
80	M	36	0.102	Medium
80	F	36	0.127	Medium
80	M	22	0.16	Medium
80	F	22	0.198	Medium
90	M	61	0.101	Medium
90	F	61	0.127	Medium
90	M	41	0.192	Medium
90	F	41	0.234	High
90	M	30	0.263	High
90	F	30	0.316	High
90	M	19	0.35	High
90	F	19	0.411	High

^*^Predicted probabilities from multiple logistic regression model using CAD4TB and demographic information that is age and gender(Model 2).

^**^Arbitrary cut-offs for TB risk (Female gender, lower age, and high CAD4TB scores associated with greater risk for TB).

## Discussion

Our study evaluated the performance of CAD4TB software as a screening tool for the detection of tuberculosis in a low-resource, high burden, non-HIV setting, using Xpert as the reference test. This study is one of the largest such evaluations of CAD4TB from a programmatic setting. In our study, CAD4TB was able to correctly identify a high proportion of people who were diagnosed with TB on Xpert and hence could potentially reduce the number of expensive molecular tests needed to detect TB in our sample of patients.

While the use of Xpert in programmatic settings has expanded in recent years, the WHO has also recommended use of more cost effective diagnostic algorithms through screening tools such as CXR^25,29,31^. Development of software that offer automated interpretation of CXRs, represents an important milestone that can link technological innovations to mass-screening programs for tuberculosis. The utilization of CAD4TB as a triage tool, to pre-screen individuals for Xpert cannot only, improve case-detection in screening programs but also possibly reduce program costs^32^.

The findings from this study indicate that CAD4TB offers high diagnostic accuracy. CAD4TB scoring can be utilized to triage individuals for Xpert testing as individuals with a low CAD4TB score had a low probability of being tested positive for TB. In resource constrained settings such as Pakistan, with limited funds to support Xpert testing for all people with presumptive TB, using a triage tool like CAD4TB could promote more rational use of Xpert by minimizing the number of cartridges used. This is also relevant for facilities where an onsite radiologist may not always be available to evaluate the CXR.f It is important to note that the savings offered through reduced Xpert tests need to be offset with the cost of acquiring and maintaining digital X-ray systems. However, a detailed discussion on the costing and policy implications for mass-screening using CXR is beyond the scope of this study. High sensitivity (range 85–97.3%) and NPV (range 96.1–98.4%) were recorded for CAD4TB at the score cut-offs utilized in the analysis, which is similar to what has been reported for CXR in other study settings^18,33,34^. The relatively lower specificity (range 30.3–65.7%) and PPV (20–30.8%) were also consistent with findings from another study evaluating CAD4TB^13^.

A high AUC (0.79) was recorded from the model using CAD4TB alone as a screening tool (Model 1). Other studies from Zambia and Bangladesh that also used Xpert as the reference test reported AUCs of 0.71 and 0.74 respectively^13,14^. Studies from Africa, using culture as the reference test reported AUC in the range 0.71–0.84^35^. Our results therefore support investigations elsewhere suggesting that CAD4TB performs well in detecting radiological abnormalities^11–14^. To date, the highest AUC has been reported with the version 3.07 of CAD4TB (compared to older versions)^35^. With newer versions available and being increasingly utilized by programmes, it is expected that a superior performance of CAD4TB software will be found in future evaluations using newer versions, with improved machine learning capacity. While the combined use of CAD4TB and symptoms has been evaluated in a previous study^12^, this is one of the first studies that have evaluated CAD4TB in combination with symptoms as well as demographic information (age and gender). Using CAD4TB in combination with demographic data enhanced the performance of the software, generating a higher AUC (0.83), while such information such as age and gender are routinely captured in screening programmes. However, including clinical symptoms to the model with demographics and CAD4TB did not significantly increase accuracy as was hypothesized by a previous study^13^. Another study from South Africa, reported a superior performance of a combination framework using both CAD4TB scores and symptoms (AUC 0.84)^12^. Symptoms may not have contributed to improved performance in our setting as the study population included individuals that were referred for investigations (including self-referrals). This may have led to pre-screening of individuals thereby limiting the added discrimination offered by symptoms. Addition of symptoms improved specificity but decreased sensitivity as a lower number of individuals would have been screened positive under Model 4, and a larger number of TB cases were missed. In order to obtain a precise estimate of the AUC and to detect differences in the AUC between the models, a large sample size was included in the study. Since the data was obtained from a programmatic setting rather than a controlled investigation, a higher proportion of MTB-ve individuals were enrolled reflecting the prevalence of the disease in this population.

The increased diagnostic accuracy offered through demographic data can be utilized to further enhance the yield for Xpert testing than through CAD4TB alone. In this study, we used the dataset to generate a range of predicted probabilities for TB detection using a combination of CAD4TB scores, age and gender, like those shown in Table 4, that can be used to devise risk categories for patients identified through screening, further refining the triage process. Our study demonstrates that for the same CAD4TB scores, variations in demographic information such as age and gender can generate different individual risk probabilities. For example, at a CAD4TB score of 80, a male aged 56 years may have a low probability (5.1%) of being identified as MTB + ve on Xpert compared to a female aged 22 years who may have a higher probability (19.8%) (Table 4). Individualized risk scores could, therefore, assist frontline healthcare workers make informed decisions about whom to test. Sputum samples for Xpert testing may be collected for those with high risk for TB, and repeat tests or clinical evaluations may be carried out for those with medium to high risk, that can potentially save Xpert cartridges, improve testing yields and make programs more cost-effective. In addition to demographic data, routinely collected programmatic information such as history of TB contact, diabetes status and smoking history can be further utilized by future programs to create personalized risk scores. It must be noted that symptoms, while not offering improved accuracy in this study, may be useful in community-settings in active case finding programs where a large number of asymptomatic individuals are also among those screened and may further help improve yield on Xpert.

Our study findings also demonstrate that for increasing CAD4TB score thresholds, the sensitivity decreased, with corresponding increase in specificity, resulting in more TB cases but providing a higher yield (Table 3). Similar findings have been reported from a study in South Africa where 11% of TB cases would have been missed using a threshold score that would have triaged 40% of suspects for Xpert testing^25^. However, individualized risk assessment, may diminish the need to set CAD4TB thresholds for programs broadly with greater reliance on testing based on personalized assessment.

An additional benefit of utilizing digital X-rays is increased capacity for clinical diagnosis of TB. Images can be archived online using cloud-based software allowing radiologists or clinical officers at TB facilities high quality images for diagnostic evaluation. In addition, mass-screening programs with X-rays are more likely to generate community interest and support mobilization than conventional screening camps with health workers. However, additional operational considerations continue to be relevant regardless of the modality of screening used. Improvements in processes such as health communication activities to promote screening among asymptomatic individuals, adequate resources for sputum induction, increased diagnostic capacity for testing, additional clinical staff for examining bacteriologically negative cases and engineers for providing equipment and software maintenance, will all be required to make screening and community referrals more effective. Since CAD4TB does not differentiate CXR abnormalities that may be observed in other conditions, such as pneumonia, lung cancer, etc., a significant number of people without TB are likely to be referred for diagnostic testing^14^. Algorithms and pathways to care will need to be developed for managing the diagnostic workup and treatment for these individuals. This is especially pertinent for developing countries with donor supported TB programs as diagnostics and treatment for other pulmonary pathologies are not funded.

Our study has certain limitations. The major limitation was that Xpert, and not mycobacterial culture was used as the reference standard, whereby Xpert negative, culture positive TB cases may have been missed. Individuals that were unable to expectorate sputum and cases with invalid or error results on xpert (for which additional sputum samples could not be obtained to re-run the test), were excluded from the study. These factors may have decreased the number of patients classified as MTB + ve and affected the accuracy of the results. An evaluation of the performance of CAD4TB compared with human readers was beyond the scope of this analysis as this has been conducted extensively in a number of studies. These evaluations utilized a combination of readings by clinical officers and radiologists and the performance of CAD4TB was found to have been comparable to those of human readers and also has the potential to reduce inter-reader and intra-reader variability and detection errors^11,34–36^. While these early studies have demonstrated the effectiveness of CAD4TB in place of medical staff, further studies such as ours that utilize a biological reference can further support the use of CAD4TB in screening programs. Finally, the external validity of our study may be limited for active-case finding programs as the participant enrollment was carried out at a facility-based setting, and the results may not be generalizable to the community setting where a large number of asymptomatic people with TB may also be present. We therefore recommend further studies to evaluate CAD4TB in the community setting such as through mobile X-ray units.

## Conclusion

This study described the first use of CXRs supported with computer-aided detection as part of enhanced case-finding intervention in the private sector in Pakistan. It demonstrated CAD4TB has the potential to be used as a triage tool to carry out screening of symptomatic individuals who could be excluded from further testing to make screening programs more cost effective by saving the number of Xpert tests. With the large scale roll-outs of Xpert and CAD4TB in local programmatic settings, its use within different case finding approaches should be evaluated and compared. A follow-up study comparing different versions of CAD4TB is also recommended. Screening algorithms need to be tailored to local contexts taking into account priorities for increased case-detection and resources required for testing additional individuals with presumptive TB.

## Data Availability

The datasets generated during and/or analysed during the current study are available from the corresponding author on reasonable request
